# *Artemisia annua* L. Polyphenols Enhance the Anticancer Effect of β-Lapachone in Oxaliplatin-Resistant HCT116 Colorectal Cancer Cells

**DOI:** 10.3390/ijms242417505

**Published:** 2023-12-15

**Authors:** Eun Joo Jung, Hye Jung Kim, Sung Chul Shin, Gon Sup Kim, Jin-Myung Jung, Soon Chan Hong, Choong Won Kim, Won Sup Lee

**Affiliations:** 1 Department of Internal Medicine, Institute of Medical Science, Gyeongsang National University Hospital, Gyeongsang National University College of Medicine, 15 Jinju-daero 816 Beon-gil, Jinju 52727, Republic of Korea; eunjoojung@gnu.ac.kr; 2 Department of Pharmacology, Institute of Medical Science, Gyeongsang National University College of Medicine, Jinju 52727, Republic of Korea; 3 Department of Chemistry, Research Institute of Life Science, Gyeongsang National University, Jinju 52828, Republic of Korea; sshin@gnu.ac.kr; 4 Research Institute of Life Science, College of Veterinary Medicine, Gyeongsang National University, Jinju 52828, Republic of Korea; gonskim@gnu.ac.kr; 5 Department of Neurosurgery, Institute of Medical Science, Gyeongsang National University Hospital, Gyeongsang National University College of Medicine, Jinju 52727, Republic of Korea; gnuhjjm@gnu.ac.kr; 6 Department of Surgery, Institute of Medical Science, Gyeongsang National University Hospital, Gyeongsang National University College of Medicine, Jinju 52727, Republic of Korea; hongsc@gnu.ac.kr; 7 Department of Biochemistry, Institute of Medical Science, Gyeongsang National University College of Medicine, Jinju 52727, Republic of Korea; cwkim@gnu.ac.kr

**Keywords:** *Artemisia annua* L. polyphenols, β-lapachone, phytochemical, anticancer effect, oxaliplatin-resistant, colorectal cancer, chemotherapy

## Abstract

Recent studies suggest that the anticancer activity of β-lapachone (β-Lap) could be improved by different types of bioactive phytochemicals. The aim of this study was to elucidate how the anticancer effect of β-Lap is regulated by polyphenols extracted from Korean *Artemisia annua* L. (pKAL) in parental HCT116 and oxaliplatin-resistant (OxPt-R) HCT116 colorectal cancer cells. Here, we show that the anticancer effect of β-Lap is more enhanced by pKAL in HCT116-OxPt-R cells than in HCT116 cells via a CCK-8 assay, Western blot, and phase-contrast microscopy analysis of hematoxylin-stained cells. This phenomenon was associated with the suppression of OxPt-R-related upregulated proteins including p53 and β-catenin, the downregulation of cell survival proteins including TERT, CD44, and EGFR, and the upregulation of cleaved HSP90, γ-H2AX, and LC3B-I/II. A bioinformatics analysis of 21 proteins regulated by combined treatment of pKAL and β-Lap in HCT116-OxPt-R cells showed that the enhanced anticancer effect of β-Lap by pKAL was related to the inhibition of negative regulation of apoptotic process and the induction of DNA damage through TERT, CD44, and EGFR-mediated multiple signaling networks. Our results suggest that the combination of pKAL and β-Lap could be used as a new therapy with low toxicity to overcome the OxPt-R that occurred in various OxPt-containing cancer treatments.

## 1. Introduction

Colorectal cancer is one of the most prevalent cancers with a high mortality rate worldwide due to high relapse after surgery and chemotherapy. Various types of dietary polyphenols derived from plants exert antioxidant, anti-atherosclerotic, anti-inflammatory, and anticancer properties, and are currently being used as a combination therapy to increase anticancer efficacy with other chemotherapeutic agents in preclinical and clinical studies for cancer prevention, cancer treatment, and drug resistance inhibition [1,2,3]. Polyphenols are heterogeneous compounds containing more than one phenol group and are mainly present in fruits and vegetables. Depending on their chemical structure and number of phenol groups, bioactive polyphenols can be divided into flavonoid compounds (e.g., flavonols, flavanols, flavones, flavanones, isoflavones, and anthocyanins) and non-flavonoid compounds (e.g., phenolic acids, hydroxycinnamic acids, lignans, stilbenes, and tannins) [4]. The anticancer mechanism of polyphenols is known to be related to the regulation of reactive oxygen species (ROS), which are involved in tumorigenesis, cancer progression, and drug resistance [5]. In addition, the anticancer mechanisms of polyphenols are associated with the modulation of various signaling pathways for cell fate determinants, such as the tumor suppressor p53, the cancer stem cell marker CD44, and the cancer prognostic marker EGFR, in a ROS-dependent or -independent manner [6,7,8,9,10].

β-Lapachone (β-Lap) is an *ortho*-naphthoquinone phytochemical isolated from the lapacho tree in the Amazon rainforest and South America, and can be synthesized from lapachol and other naphthoquinone metabolites; it exerts various bioactivities such as antioxidant, anti-inflammatory, and anticancer effects with low toxicity, and is being used in preclinical and phase II clinical trials in the USA for the treatment of advanced solid tumors [11,12,13,14]. It is known that the enzyme NAD(P)H:quinone oxidoreductase (NQO1) is predominantly required for apoptotic or necrotic cell death induced by β-Lap in various cancer cell types through the modulation of DNA damage, caspase-3, PARP1, PI3K/AKT signaling, NF-κB, telomerase reverse transcriptase (TERT), DNA topoisomerase I and II, and CD44 [15,16,17,18,19,20,21,22,23]. In addition, autophagic cell death was induced by β-Lap in U87 MG glioma cells through an increase in ROS, LC3-I to LC3-II conversion, and acidic vesicular organelles [24]. Notably, β-Lap treatment alone induced cell cycle arrest and apoptosis in human leukemia, prostate, and colon cancer cells in a p53-independent manner [25,26], and the combined treatment of β-Lap and paclitaxel was shown to induce synergistic apoptosis in human retinoblastoma Y79 cells through the downregulation of phospho-AKT levels and upregulation of p53 [27]. However, synergistic apoptosis induced by the combined treatment of β-Lap and ionizing radiation in RKO human colon adenocarcinoma cells was associated with the downregulation of p53, p21, and cyclin B1/Cdc2 kinase activity [28]. These results suggest that the main anticancer mechanism of β-Lap associated with p53 is still unclear and may differ depending on the cell type and combined chemotherapeutic agent.

Oxaliplatin (OxPt) is a commonly used chemotherapeutic agent in metastatic colorectal cancer treatment and is used as a combined therapy with different types of drugs [29,30]. Unfortunately, OxPt-containing therapeutics cause drug resistance and adverse effects, ultimately leading to treatment failure. To find a new mechanism for OxPt resistance (OxPt-R), we previously generated OxPt-resistant HCT116 colorectal cancer cells, named HCT116-OxPt-R cells, and revealed that the anticancer activity by β-Lap in HCT116-OxPt-R cells was related to the downregulation of OxPt-R-related upregulated and functionally inactivated p53 [31]. In this study, to find a new mechanism to improve the anticancer mechanism of β-Lap, we first examined whether the anticancer effect of β-Lap could be enhanced by polyphenols isolated from Korean *Artemisia annua* L. (pKAL) in parental HCT116 and HCT116-OxPt-R cells. Next, we compared the anticancer effect and mechanism on the combined treatment of pKAL and β-Lap between HCT116 and HCT116-OxPt-R cells. Finally, gene ontology and string analysis were performed to better understand the potential roles of the proteins involved in enhancing the anticancer activity of β-Lap by pKAL in HCT116-OxPt-R cells. As a result, we found the following 21 proteins regulated by the combined treatment of pKAL and β-Lap in HCT116-OxPt-R cells: p53, β-catenin, PLCγ1, ERK, NF-κB p65, survivin, cyclin B1, cyclin A, TERT, CD44, EGFR, cyclin D1, caspase-8, PARP1, beclin-1, AKT, LDHA, PCNA, HSP90, γ-H2AX, and LC3B-I/II. Collectively, we show here that the anticancer effect of β-Lap can be enhanced more effectively by pKAL in HCT116-OxPt-R cells than in HCT116 cells by promoting the modulation of 21 proteins; this occurs, especially, through the downregulation of TERT, CD44, EGFR, and OxPt-R-related upregulated proteins including p53 and β-catenin.

## 2. Results

### 2.1. Anticancer Effect by Combined Treatment of pKAL and β-Lap in HCT116 Cells

**Figure 1:**
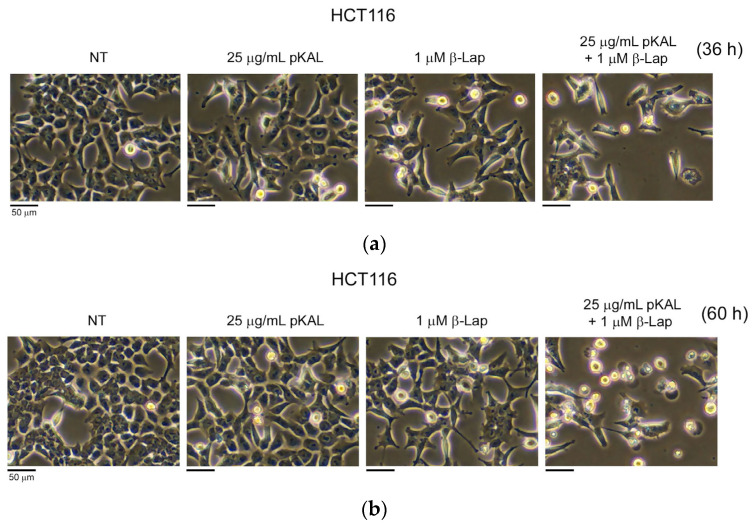
Regulation of cell morphology by combined treatment of pKAL and β-Lap in HCT116 cells: HCT116 cells were grown for 20 h on a 10 cm culture dish and then non-treated (NT) or treated with the indicated amounts of pKAL and β-Lap for 36 h (**a**) or 60 h (**b**). Cell morphology was analyzed via phase-contrast microscopy.

First, we investigated whether the anticancer effects of pKAL and β-lap could be enhanced by combined treatment compared to singular treatment in HCT116 cells. Cells were treated with 25 μg/mL pKAL alone, 1 μM β-Lap alone, or a combination of 25 μg/mL pKAL and 1 μM β-Lap for 36 and 60 h, and then morphological changes were analyzed via phase-contrast microscopy. As shown in Figure 1a, the combined treatment of pKAL and β-Lap for 36 h resulted in a decrease in cell number and significant morphological changes compared to single treatment. This phenomenon was more significantly induced in the 60 h treatment, and many round cells with an altered intracellular structure were detected in the combined treatment of pKAL and β-Lap, but not significantly in the less toxic 25 μg/mL pKAL or 1 μM β-Lap (Figure 1b). These results show that the anticancer activity of pKAL and β-Lap in HCT116 cells can be enhanced more effectively by combined treatment than by singular treatment.

**Figure 2:**
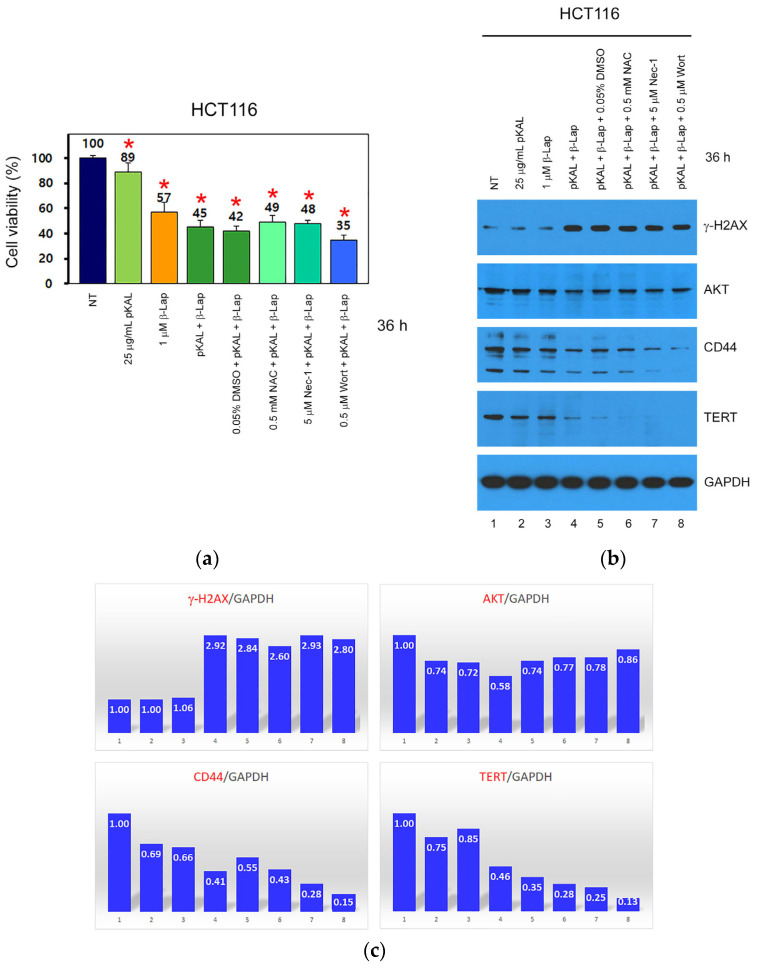
Effect of NAC, Nec-1, and Wort on the regulation of cell viability and protein levels via the combined treatment of pKAL and β-Lap in HCT116 cells. HCT116 cells were grown for 20 h on a 24-well plate (**a**) or 10 cm dish (**b**) and then treated with the indicated amounts of drugs for 36 h. (**a**) Cell viability was analyzed via a CCK-8 assay in two triplicate tests. Statistical significance between control and sample was determined using Student’s *t*-test, * *p* < 0.05; (**b**) whole cell extracts were prepared using 1x SDS sample buffer and analyzed via Western blot using the indicated antibodies; (**c**) densitometry analysis of protein bands was conducted in the panels of Figure 2b using the ImageJ program (version 1.53k).

Next, we elucidated whether enhanced anticancer activity via the combined treatment of pKAL and β-Lap in HCT116 cells is related to ROS, necroptosis, and PI3K signaling. Cells were treated for 36 h with 25 μg/mL pKAL, 1 μM β-Lap, or a combination of 25 μg/mL pKAL and 1 μM β-Lap in the absence or presence of N-acetyl-L-cysteine (NAC), necrostatin-1 (Nec-1), or wortmannin (Wort), which are inhibitors of ROS, necroptosis, and PI3K, respectively, and then cell viability was analyzed via a CCK-8 assay. As expected, the downregulation of cell viability via a combined treatment of pKAL and β-Lap (45%) was higher compared to treatment with 25 μg/mL pKAL (89%) or 1 μM β-Lap (57%) alone. In addition, the downregulation of cell viability via combined treatment of pKAL and β-Lap in the presence of 0.05% DMSO control solvent (42%) was slightly inhibited by 0.5 mM NAC (49%) and 5 μM Nec-1 (48%), respectively, but it was somewhat promoted by 0.5 μM Wort (35%) (Figure 2a). To better understand these results, the protein levels of γ-H2AX, AKT, CD44, and TERT were examined using Western blot and densitometry analysis. As a result, enhanced anticancer activity via the combined treatment of pKAL and β-Lap was significantly associated with the upregulation of γ-H2AX, a DNA damage marker, and the downregulation of cell survival proteins such as AKT, CD44, and TERT (Figure 2b, lanes 1–4; Figure 2c, bar graphs 1–4). Notably, the downregulation of CD44 and TERT via combined treatment of pKAL and β-Lap was promoted by NAC, Nec-1, or Wort (Figure 2b, lanes 5–8; Figure 2c, bar graphs 5–8). Therefore, these results show that the enhanced anticancer effect of pKAL and β-Lap combined treatment is associated with the regulation of ROS, necroptosis, and PI3K signaling. However, the inhibition of ROS and necroptosis-mediated cell death signaling by NAC and Nec-1 may not be effective due to the downregulation of cell survival proteins such as CD44 and TERT. And, the inhibition of PI3K-mediated cell survival signaling by Wort may be ineffective due to the downregulation of DNA damage marker γ-H2AX.

### 2.2. Anticancer Effect by Combined Treatment of pKAL and β-Lap in HCT116-OxPt-R Cells

**Figure 3:**
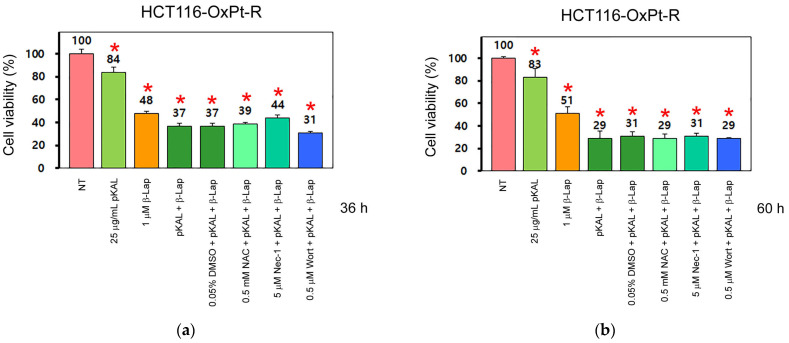

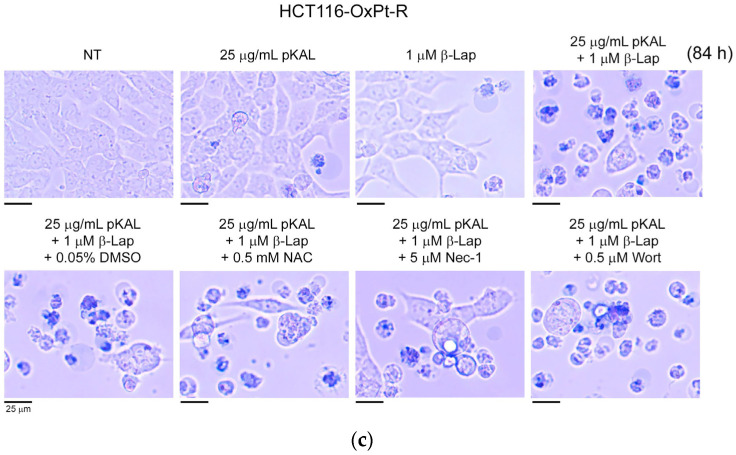
Effect of NAC, Nec-1, and Wort on the regulation of cell viability and dead cells via combined treatment of pKAL and β-Lap in HCT116-OxPt-R cells. HCT116-OxPt-R cells were grown for 20 h on a 24-well (**a**,**b**) or 6-well plate (**c**) and then treated with the indicated amounts of drugs for 36 h (**a**), 60 h (**b**), or 84 h (**c**). (**a**,**b**) Cell viability was analyzed via CCK-8 assay in two triplicate tests. Statistical significance was determined using Student’s *t*-test, * *p* < 0.05; (**c**) dead cells were analyzed using phase-contrast microscopy after trypan blue staining.

Based on the treatments applied to the HCT116 cells, we elucidated whether the anticancer effects of pKAL and β-lap could be enhanced by combined treatment compared to single treatment in HCT116-OxPT-R cells, and whether this phenomenon was related to ROS, necroptosis, and PI3K signaling. As shown in Figure 3a, the downregulation of cell viability via the combined treatment of pKAL and β-Lap (37%) for 36 h was higher than with the singular treatment of 25 μg/mL pKAL (84%) or 1 μM β-Lap (48%). In addition, the downregulation of cell viability via a combined treatment of pKAL and β-Lap in the presence of 0.05% DMSO (37%) was somewhat inhibited by 5 μM Nec-1 (44%), but not significantly by 0.5 mM NAC (39%), whereas it was slightly increased by 0.5 μM Wort (31%). Moreover, the downregulation of cell viability by a combined treatment of pKAL and β-Lap was higher in the 60 h treatment (29%) than in the 36 h treatment (37%); however, treatment of pKAL (83%) or β-Lap (51%) with a single agent for 60 h was not significantly changed compared to 36 h of singular treatment of pKAL (84%) or β-Lap (48%) (compare Figure 3a,b). Notably, the downregulation of cell viability via the combined treatment of pKAL and β-Lap in the presence of 0.05% DMSO (31%) for 60 h was not significantly affected by 0.5 mM NAC (29%), 5 μM Nec-1 (31%), or 0.5 μM Wort (29%) (Figure 3b). Consistent with these results, dead cells stained with trypan blue were remarkably induced via the combined treatment of pKAL and β-Lap for 84 h, but not significantly via the single treatment of pKAL or β-Lap (Figure 3c, upper panels). In addition, the induction of dead cells caused by the combined treatment of pKAL and β-Lap for 84 h was not significantly affected by 0.5 mM NAC, 5 μM Nec-1, or 0.5 μM Wort (Figure 3c, lower panels). Collectively, these results suggest that the enhanced anticancer effect by the combined treatment of pKAL and β-Lap for 36 h may be associated with necroptosis and downregulation of PI3K signaling in HCT116-OxPt-R cells, but may not be significantly related to ROS production; however, this phenomenon may be altered by the long-term combined treatment of pKAL and β-Lap through an unknown mechanism.

### 2.3. Comparison of Anticancer Effects between HCT116 and HCT116-OxPt-R Cells by Combined Treatment of pKAL and β-Lap

**Figure 4:**
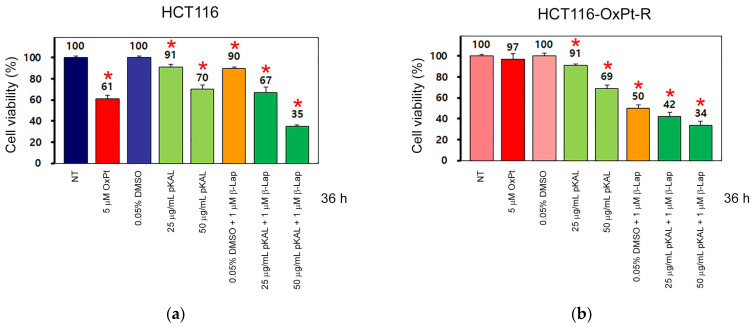
Comparison of cell viability effect by combined treatment of pKAL and β-Lap between HCT116 and HCT116-OxPt-R cells. HCT116 (**a**) and HCT116-OxPt-R cells (**b**) were grown for 20 h on a 24-well plate and then treated with the indicated amounts of drugs for 36 h. Cell viability was analyzed using a CCK-8 assay in triplicate tests. Statistical significance between control and sample was determined using Student’s *t*-test, * *p* < 0.05.

To better understand the anticancer effects induced by the combined treatment of pKAL and β-Lap, HCT116 and HCT116-OxPt-R cells were treated with 5 μM OxPt, 25 μg/mL pKAL, 50 μg/mL pKAL, or a combination of 1 μM β-Lap and 0.05% DMSO; 1 μM β-Lap and 25 μg/mL pKAL; and 1 μM β-Lap and 50 μg/mL pKAL for 36 h. The cell viability was compared between these cells via a CCK-8 assay. As a result, cell viability was downregulated by 5 μM OxPt in HCT116 cells (61%), but not significantly in HCT116-OxPt-R cells (97%). The downregulation of cell viability following pKAL treatment alone was not significantly different between HCT116 and HCT116-OxPt-R-cells (Figure 4a,b, compare bar graphs 3–5). Notably, the downregulation of cell viability by β-Lap in the presence of 0.05% DMSO was significantly higher in HCT116-OxPt-R cells (50%) than in HCT116 cells (90%). Moreover, the downregulation of cell viability by β-Lap was increased by pKAL in both HCT116 and HCT116-OxPt-R cells in a pKAL concentration-dependent manner (Figure 4a,b, compare bar graphs 6–8). These results show that the higher anticancer activity from the combined treatment of pKAL and β-Lap in HCT116-OxPt-R cells compared to HCT116 cells may be due to the higher anticancer activity of β-Lap in HCT116-OxPt-R cells than in HCT116 cells.

**Figure 5:**
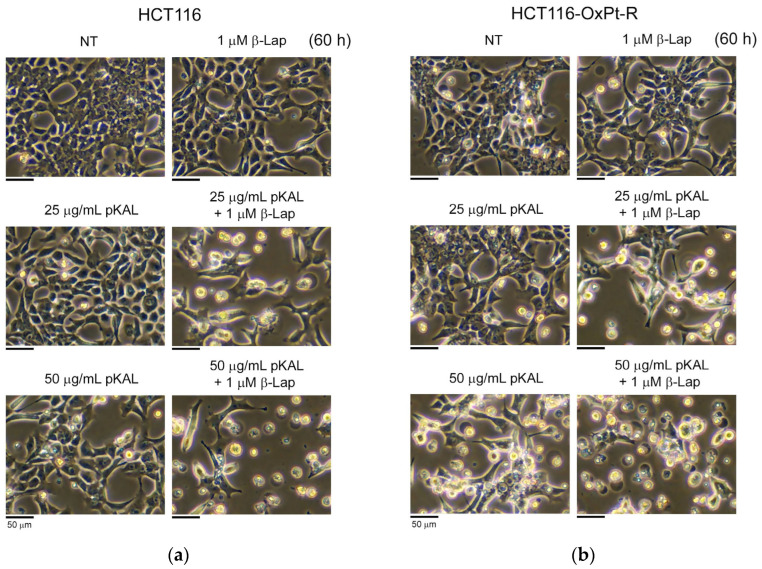
Effect of cell morphological changes by pKAL and β-Lap in HCT116 and HCT116-OxPt-R cells. HCT116 (**a**) and HCT116-OxPt-R cells (**b**) were grown for 20 h on a 10 cm dish and then treated with the indicated amounts of drugs for 60 h. Cell morphology was analyzed using phase-contrast microscopy.

To further elucidate the anticancer effect induced by the combined treatment of pKAL and β-Lap, HCT116 and HCT116-OxPt-R cells were treated with 25 μg/mL pKAL, 50 μg/mL pKAL, 1 μM β-Lap, or a combination of 25 μg/mL pKAL and 1 μM β-Lap, and 50 μg/mL pKAL and 1 μM β-Lap for 60 h, and morphological changes between these cells were compared using phase-contrast microscopy. As shown in Figure 5a,b, cell morphology was significantly altered by 50 μg/mL pKAL in both cells, but not significantly changed by the less toxic 25 μg/mL pKAL (left panels). In addition, the morphological changes with the 1 μM β-Lap treatment alone were somewhat higher in HCT116-OxPt-R cells than in HCT116 cells, and the morphological changes with the combined treatment of pKAL and β-Lap were significantly higher than with the singular treatment in both cells (right panels). The effect of cell viability following 50 μg/mL pKAL treatment for 36 h showed no significant difference between HCT116 and HCT116-OxPt-R cells (Figure 4a,b), but the morphological changes following 50 μg/mL pKAL treatment for 60 h were significantly higher in HCT116-OxPt-R cells than in HCT116 cells (Figure 5a,b). These results suggest that the anticancer effect caused by pKAL may be maintained longer in HCT116-OxPt-R cells than in HCT116 cells.

**Figure 6:**
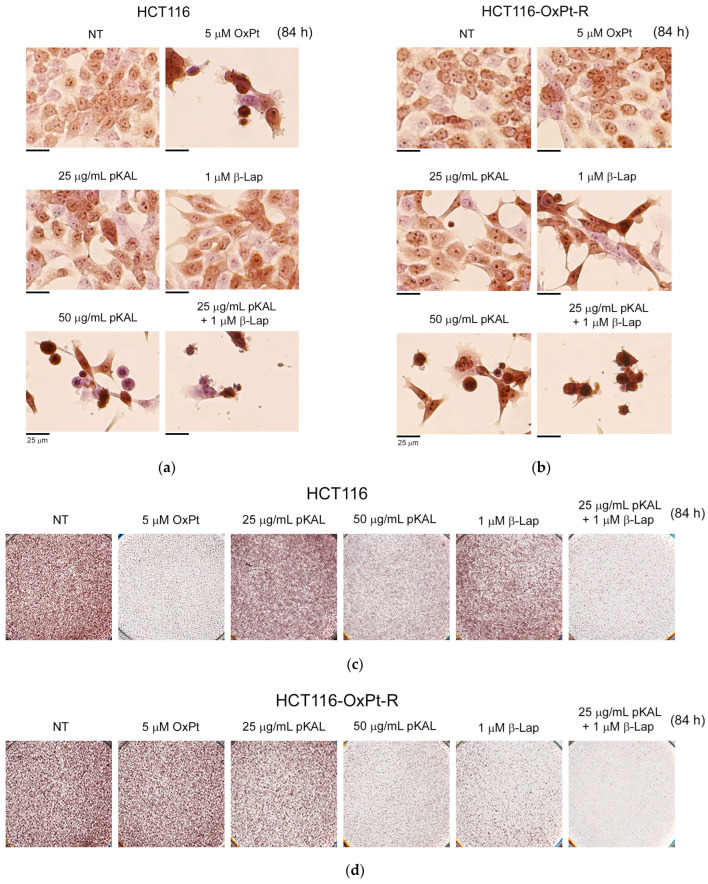
Effect of pKAL and β-Lap on the regulation of nuclear morphology and cell survival in HCT116 and HCT116-OxPt-R cells. HCT116 (**a**,**c**) and HCT116-OxPt-R cells (**b**,**d**) were grown for 20 h on a 6-well plate and then treated with the indicated amounts of drugs for 84 h. (**a**,**b**) Cell morphology was analyzed using phase-contrast microscopy after hematoxylin staining. (**c**,**d**) Attached cells on the 6-well plate were analyzed via scanning after the phase-contrast microscopy of hematoxylin-stained cells.

To further elucidate the morphological changes induced by the long-term treatment of pKAL and β-Lap, HCT116 and HCT116-OxPt-R cells were treated with 25 μg/mL pKAL, 50 μg/mL pKAL, 5 μM OxPt, 1 μM β-Lap, or a combination of 25 μg/mL pKAL and 1 μM β-Lap for 84 h on a 6-well dish, and then the cells were stained with hematoxylin solution and analyzed using phase-contrast microscopy. As shown in Figure 6a,b, cell morphology was significantly altered by 5 μM OxPt in HCT116 cells, but not significantly in HCT116-OxPt-R cells. In addition, cell morphology was significantly altered by 50 μg/mL pKAL in both cells, but not significantly changed by the less toxic 25 μg/mL pKAL (left panels). The morphological changes caused by 1 μM β-Lap treatment alone were significantly higher in HCT116-OxPt-R cells than in HCT116 cells, and the morphological changes following the combined treatment of pKAL and β-Lap were significantly higher than following the singular treatment in both cells (right panels). After a phase-contrast microscopy analysis of hematoxylin-stained cells, 6-well dishes were scanned, and the images are shown in Figure 6c,d. These results suggest that the anticancer effect of 1 μM β-Lap can be maintained for a longer time in HCT116-OxPt-R cells compared to HCT116 cells, and this phenomenon is the main reason for the higher anticancer activity following the combined treatment of pKAL and β-Lap in HCT116-OxPt-R cells compared to HCT116 cells.

### 2.4. Anticancer Mechanism of Combined Treatment of pKAL and β-Lap in HCT116 and HCT116-OxPt-R Cells

**Figure 7:**
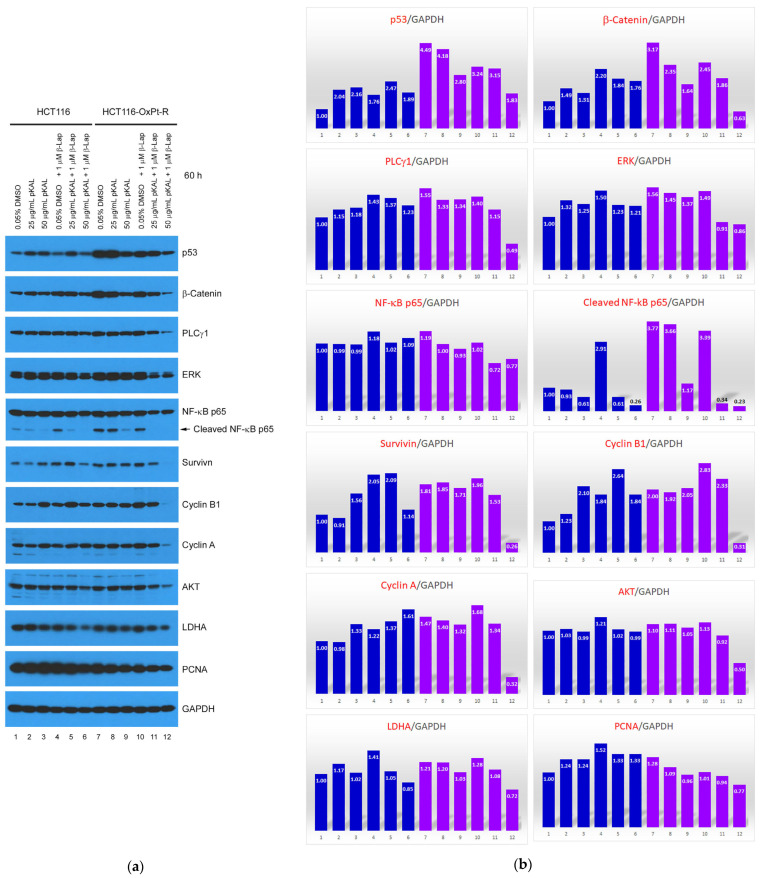

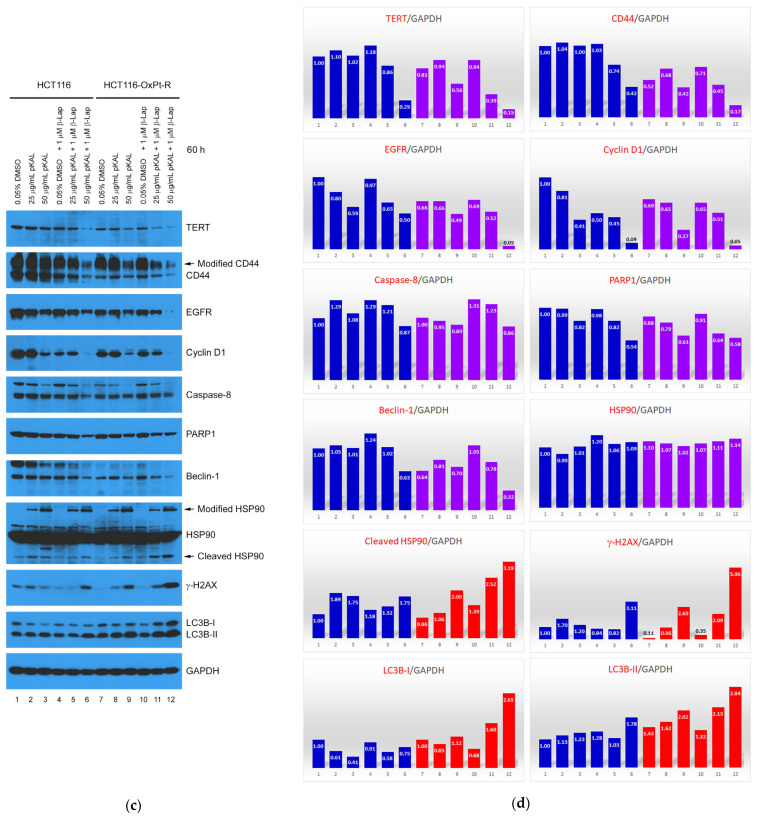
Regulation of protein levels by pKAL and β-Lap in HCT116-OxPt-R cells. (**a**,**c**) HCT116 and HCT116-OxPt-R cells were grown for 20 h on a 10 cm dish and then treated with the indicated amounts of drugs for 60 h. Whole cell extracts were prepared using 1x SDS sample buffer and analyzed via Western blot using the indicated antibodies. (**b**,**d**) Densitometry analysis of protein bands in the panels of (**a**,**c**) using the ImageJ program (version 1.53k).

To identify a novel molecular mechanism related to the enhanced anticancer effects following the combination treatment of pKAL and β-Lap, Western blot analysis was performed using various antibodies involved in cell survival and death signaling in whole cell extracts of HCT116 and HCT116-OxPt-R cells, and the resulting protein bands were quantified using the ImageJ program (version 1.53k). As shown in Figure 7a,b, the OxPt-R properties in HCT116-OxPt-R cells were associated with the significant upregulation of p53, β-catenin, PLCγ1, ERK, cleaved NF-κB p65, survivin, cyclin B1, and cyclin A, and somewhat upregulation of NF-κB p65, AKT, LDHA, and PCNA (compare lanes 1,7). Except for cyclin B1, all of the upregulated proteins in HCT116-OxPt-R cells were downregulated by pKAL, but this phenomenon appeared to be differently regulated in HCT116 cells (compare lanes 1–3 and 7–9). Moreover, OxPt-R-related upregulated proteins, except for p53, β-catenin, and PCNA, were not significantly downregulated following the treatment of the less toxic 1 μM β-Lap in HCT116-OxPt-R cells, whereas all of the OxPt-R-related upregulated proteins were significantly downregulated following the combined treatment of pKAL and β-Lap in HCT116-OxPt-R cells in an almost pKAL concentration-dependent manner, and this phenomenon more effectively occurred in HCT116-OxPt-R cells than in HCT116 cells (compare lanes 4–6 and 10–12).

Consistent with the results of Figure 2b, the downregulation of TERT and CD44 was associated with an enhanced anticancer effect via the combined treatment of pKAL and β-Lap in HCT116 cells in a pKAL concentration-dependent manner (Figure 7c,d, lanes 1–6). Notably, TERT, CD44, EGFR, cyclin D1, PARP1, and beclin-1 were suppressed in HCT116-OxPt-R cells (Figure 7c,d, compare lanes 1,7), and EGFR, cyclin D1, and PARP1 were downregulated by pKAL in a concentration-dependent manner in both HCT116 and HCT116-OxPt-R cells (Figure 7c,d, lanes 1–3, 7–9). Moreover, cyclin D1 was significantly downregulated by β-Lap in HCT116 cells, but not in HCT116-OxPt-R cells, suggesting that the suppression of cyclin D1 is involved in the anticancer mechanism of β-Lap in HCT116 cells but not in HCT116-OxPt-R cells (Figure 7c,d, compare lanes 1,4 and 7,10). Importantly, the enhanced anticancer effect of β-Lap by pKAL was significantly associated with the downregulation of TERT, CD44, EGFR, cyclin D1, caspase-8, PARP1, and beclin-1 in both HCT116 and HCT116-OxPt-R cells in a pKAL concentration-dependent manner, and the downregulation of TERT, CD44, EGFR, and beclin-1 was promoted more effectively in HCT116-OxPt-R-cells than in HCT116 cells (Figure 7c,d, compare lanes 4–6 and 10–12). In contrast, cleaved HSP90, γ-H2AX, and LC3B-I/II were significantly upregulated via the combined treatment of pKAL and β-Lap in HCT116-OxPt-R cells compared with HCT116 cells (Figure 7c,d, compare lanes 1–6 and 7–12).

Collectively, these results show that pKAL enhances the anticancer effect of β-Lap in both HCT116 and HCT116-OxPt-R cells using a somewhat different mechanism. In addition, pKAL enhances the anticancer effect of β-Lap more effectively in HCT116-OxPt-R cells than in HCT116 cells. In particular, pKAL enhances the anticancer effect of β-Lap through the downregulation of cell survival proteins including TERT, CD44, and EGFR, the suppression of OxPt-R-related upregulated proteins including p53 and β-catenin, and the upregulation of cell death processes including cleaved HSP90, γ-H2AX, and LC3B-I/II.

### 2.5. Bioinformatics Analysis for21 Proteins Regulated by Combined Treatment of pKAL and β-Lap in HCT116-OxPt-R Cells

To better understand the enhanced anticancer mechanism of β-Lap by pKAL in HC116-OxPt-R cells, we searched the gene symbols and keywords for 21 proteins modulated via the combined treatment of pKAL and β-Lap through the UniProt bioinformatics database (https://www.uniprot.org) (accessed on 10 September 2023), and these are shown in Table 1.

Using the 21 gene symbols, namely AKT1, BECN1, BIRC5, CASP8, CCNA1, CCNB1, CCND1, CD44, CTNNB1, EGFR, H2AX, HSP90AA1, LDHA, MAP1LC3B, MAPK3, PARP1, PCNA, PLCG1, RELA, TERT, and TP53, we performed a gene ontology analysis through the DAVID bioinformatics database (http://david.ncifcrf.gov/tools.jsp) (accessed on 10 September 2023), and the results are shown in Table 2. Thus, the results of Figure 7 and Table 2 suggest that the enhanced anticancer mechanism of β-Lap by pKAL in HCT116-OxPt-R cells may be significantly associated with the regulation of various biological processes such as the negative regulation of apoptotic process via the downregulation of beclin-1, β-catenin, survivin, AKT1, p53, CD44, NF-κB p65, and EGFR; positive regulation of protein phosphorylation via the upregulation of cleaved HSP90 and downregulation of cyclin D1, survivin, AKT1, EGFR, and ERK1; cellular response to epidermal growth factor stimulus via the downregulation of beclin-1, AKT1, PLCγ1, and EGFR; positive regulation of G1/S transition of mitotic cell cycle via the downregulation of cyclin D1, TERT, AKT1, and EGFR; and positive regulation of pre-miRNA transcription from RNA polymerase II promoter via the downregulation of TERT, p53, NF-kB p65, and EGFR. In addition, the results of Figure 7 and Table 2 suggest that the enhanced anticancer mechanism of β-Lap by pKAL in HCT116-OxPt-R cells may be associated with the regulation of the following biological processes: regulation of apoptotic process via the upregulation of cleaved HSP90 and downregulation of caspase-8, survivin, AKT1, and p53; response to xenobiotic stimulus via upregulation of cleaved HSP90 and downregulation of beclin-1, cyclin D1, β-catenin, and p53; cellular response to DNA damage stimulus via the upregulation of γ-H2AX and downregulation of cyclin D1, PARP1, p53, and ERK1; macromitophagy via the upregulation of LC3B-I/II and downregulation of beclin-1 and p53; and mitotic cell cycle phase transition via the downregulation of cyclin A1, cyclin B1, and cyclin D1.

**Figure 8:**
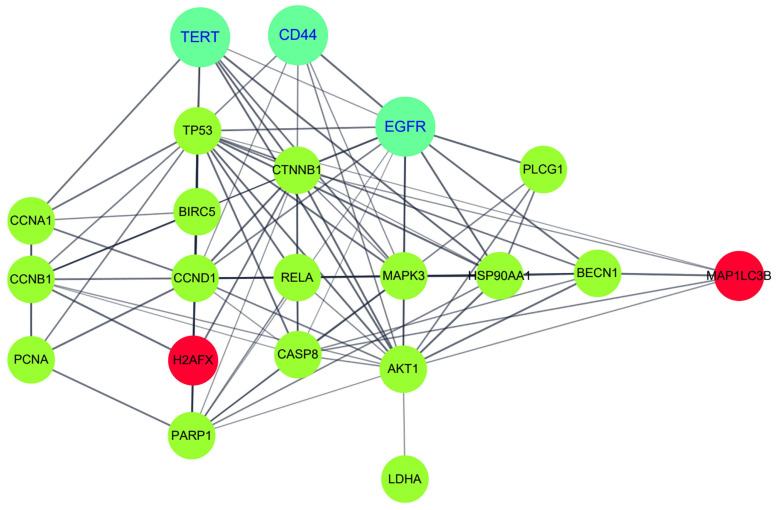
String analysis for proteins associated with the enhanced anticancer effect of β-Lap by pKAL in HCT116-OxPt-R cells. Protein–protein interaction networks were analyzed via string analysis using the Cytoscape software (v.3.7.1.). Downregulated and upregulated proteins are shown in green and red, respectively, with their gene symbols.

To further understand the enhanced anticancer mechanism of β-Lap by pKAL in HC116-OxPt-R cells, the potential protein–protein interaction network for the 21 proteins was analyzed via a string analysis using the Cytoscape software (http://www.cytoscape.org) (accessed on 10 September 2023). As shown in Figure 8, the enhanced anticancer effect of β-Lap by pKAL was associated with the modulation of multiple protein–protein interaction signaling networks connected by TERT/p53/survivin/cyclin D1/H2AX/PARP1 signaling; CD44/β-catenin/NF-κB p65/caspase-8 signaling; EGFR/ERK1/AKT1/LDHA signaling; TERT/cyclin A1/cyclin B1/PCNA signaling; EGFR/HSP90/beclin-1/LC3B signaling, etc.

Taken together, we show that pKAL enhances the anticancer effect of β-Lap in HCT116-OxPt-R colorectal cancer cells through the modulation of TERT, CD44, and EGFR-mediated multiple signaling networks.

## 3. Discussion

In order to overcome OxPt-R, a new combination therapy that is less cytotoxic and can induce anticancer activity more efficiently is needed to replace the OxPt-containing combination treatments that have developed oxaliplatin resistance. In this study, our results showed that the anticancer activity of β-Lap was more effectively enhanced by pKAL in HCT116-OxPt-R cells than in parental HCT116 cells, and the related anticancer mechanism was revealed to better understand the combined treatment effects of pKAL and β-Lap.

*Artemisia annua* L. (AL), a plant belonging to the Asteraceae family, has been traditionally used for the treatment of malaria in Asia and Africa as a tea or press juice, and it has many biological activities such as anti-inflammatory, anticancer, immunoregulatory, anti-adipogenic, anti-ulcerogenic, and anti-asthmatic [32,33]. Studies on the biological activities of AL were mainly accomplished using artemisinin isolated from AL, and artemisinin and its derivatives have been shown to exhibit anticancer activity in various types of cancer cells and in clinical trials for the treatment of prostate carcinoma by modulating DNA damage and tumor biomarkers [34,35,36,37]. In addition, artemisinin and its derivatives (e.g., artesunate and dihydroartemisinin) have been studied in combination therapy with other standard anticancer drugs, radiation, and natural products to improve anticancer activity [33]. However, the artemisinin-deficient AL extract isolated via acetonitrile maceration showed strong anticancer activity in MDA-MB-231 human breast cancer cells, and the most abundant components of the extract were identified as chrysosplenol D, arteannuin B, and casticin [38]. Therefore, further studies on the unknown bioactive compounds in AL are needed to better understand the anticancer properties of AL.

It is known that natural polyphenols isolated from plants, such as curcumin, resveratrol, quercetin, kaempferol, luteolin, EGCG, and apigenin, have a promising chemotherapeutic potential for colorectal cancer therapy [2,39]. In addition, the extracts of polyphenols isolated from apple and AL were shown to have a potential ability to inhibit cancer metastasis by inhibiting epithelial–mesenchymal transition (EMT), migration, adhesion, and invasion in MDA-MB-231 triple-negative breast cancer cells [40,41]. Moreover, recent studies suggest that combination therapy using polyphenols with different types of chemotherapeutic agents could be an efficient way to improve anticancer activity and inhibit drug resistance [42].

We have previously shown that pKAL exerts anticancer activity in both parental MDA-MB-231 and radiation-resistant MDA-MB 231 cells by downregulating CD44, Oct3/4, β-catenin, and MMP-9 [43]. In this study, we showed that a remarkable dead cell morphology was detected by the combined treatment of 25 μg/mL pKAL and 1 μM β-Lap for 60 h in HCT116 and HCT116-OxPt-R cells, but not by single treatment of 25 μg/mL pKAL or 1 μM β-Lap (Figure 1 and Figure 5). However, cell morphology was significantly changed with 50 μg/mL pKAL treatment alone, which resulted in higher cytotoxicity and morphological changes than 25 μg/mL pKAL treatment (Figure 4, Figure 5 and Figure 6). Thus, the anticancer effect with the combined treatment of 50 μg/mL pKAL and 1 μM β-Lap was higher than that seen with the combined treatment of 25 μg/mL pKAL and 1 μM β-Lap (Figure 4, Figure 5 and Figure 6). However, it seems to be more important to reveal a synergistic effect via combined treatment with less toxic amounts to reduce cytotoxicity in preclinical and clinical studies. Interestingly, a synergistic anticancer effect using a combined treatment of less toxic 25 μg/mL pKAL and 1 μM β-Lap for 84 h was remarkably detected via phase-contrast microscopy after hematoxylin staining in both cells (Figure 6). Moreover, the anticancer effect of β-Lap was higher in HCT116-OxPt-R cells than in HCT116 cells, which was associated with higher anticancer activity following the combined treatment of pKAL and β-Lap in HCT116-OxPt-R cells than in HCT116 cells (Figure 4, Figure 5 and Figure 6).

We identified 21 proteins regulated via the combined treatment of pKAL and β-Lap in HCT116-OxPt-R cells via Western blot and densitometry analysis (Figure 7). The results showed that the enhancement of the anticancer activity of β-Lap by pKAL was associated with the suppression of upregulated proteins in HCT116-OxPt-R cells, such as p53, β-catenin, PLCγ1, ERK, NF-κB p65, cleaved NF-κB p65, survivin, cyclin B1, cyclin A, AKT, LDHA, and PCNA (Figure 7a,b). In addition, it was associated with the downregulation of TERT, CD44, EGFR, cyclin D1, caspase-8, PARP1, and beclin-1, whereas it was associated with the upregulation of cleaved HSP90, γ-H2AX, and LC3B-I/II (Figure 7c,d). However, the anticancer mechanism of pKAL was somewhat different between HCT116 and HCT116-OxPt-R cells, probably due to the significant upregulation of functionally inactivated p53, β-catenin, PLCγ1, ERK, and cleaved NF-κB p65 (Figure 7a,b, compare lanes 1–3 and 7–9). In addition, our results showed that the anticancer activity of β-Lap was significantly higher in HCT116-OxPt-R cells than in HCT116 cells probably due to the downregulation of p53, β-catenin, and PCNA (Figure 4, Figure 5 and Figure 6; Figure 7a,b, compare lanes 1, 4 and 7, 10). Gene ontology and string analysis for 21 proteins regulated via the combined treatment of pKAL and β-Lap showed that the enhanced anticancer effect of β-Lap by pKAL was related to the inhibition of negative regulation of the apoptotic process and the induction of DNA damage through multiple signaling networks mediated by TERT, CD44, and EGFR (Table 2 and Figure 8).

Taken together, our results show that pKAL enhances the anticancer effect of β-Lap in HCT116 colorectal cancer cells containing OxPt-R by downregulating TERT, CD44, EGFR, and upregulated oxaliplatin resistance-related proteins. We suggest that the combination of phytochemicals such as pKAL and β-Lap could be an efficient and less toxic therapy to overcome OxPt-R caused by long-term treatment with OxPt-containing chemotherapeutic agents in various types of cancer.

## 4. Materials and Methods

### 4.1. Materials

β-lapachone synthesized by Mazence Inc. (Suwon, Republic of Korea) was kindly provided by Prof. Gi Ryang Kweon (Chungnam National University School of Medicine, Daejeon, Republic of Korea). Oxaliplatin (Eloxatin) was from Sanofi-Aventise Inc. (Seoul, Republic of Korea). Penicillin–Streptomycin (10,000 U/mL) and TrypLE^TM^ Express Enzyme with phenol red were from Thermo Fisher Scientific (Grand Island, NY, USA). RPMI 1640 medium was from HyClone (Logan, UT, USA). N-Acetyl-L-cysteine (NAC), necrostatin-1 and wortmannin were from Sigma-Aldrich (St. Louis, MO, USA). Hematoxylin solution and formaldehyde solution (4%) were obtained from Merck KGaA (Darmstadt, Germany). Cell counting kit-8 (CCK-8) was obtained from Dojindo (Kumamoto, Japan). Hematoxylin solution and formaldehyde solution (4%) were obtained from Merck KGaA (Darmstadt, Germany). Trypan blue 0.4% solution was obtained from Bioworld (Louis Park, MN, USA). Protein assay dye reagent concentrate and 30% acrylamide/bis solution 29:1 were obtained from Bio-Rad (Hercules, CA, USA). Tween-20 and DMSO were obtained from Amresco (Solon, OH, USA). The 0.22 μM nitrocellulose (NC) transfer membrane was obtained from GVS Life Sciences (Sanford, ME, USA). ECL Ottimo Western blot detection kit was obtained from TransLab (Daejeon, Republic of Korea). X-ray film (CP-BU NEW) was obtained from AGFA (Mortsel, Belgium). Dishes, plates, tubes, and pipettes for cell culture were obtained from SPL Life Sciences (Pocheon, Republic of Korea) or Thermo Fisher Scientific (Rockford, IL, USA). Akt1/2/3 (H-136), BECN1/Beclin-1 (G-11), β-catenin (E-5), caspase-8 (8CSP03), cyclin-A (H-432), cyclin-B1 (GNS1), cyclin-D1 (A-12), EGFR (1005)-G, ERK1 (K-23), HSP90α/β (H-114), NFκB-p65 (F-6), p53 (DO-1), PARP1 (F-2), PCNA (FL-261), PLC-γ1 (E-12), survivin (D-8), TERT (A-6), and GAPDH (FL-335) antibodies were obtained from Santa Cruz Biotechnology (Santa Cruz, CA, USA). CD44 (EPR1013Y) and LC3B (ab51520) antibodies were obtained from Abcam Biotechnology (Cambridge, United Kingdom). Phospho-Ser139-H2AX (γ-H2AX) antibody was obtained from Upstate Biotechnology (Lake Placid, NY, USA). LDHA was obtained from Cell Signaling Technology (Beverly, MA, USA). Secondary goat anti-rabbit and anti-mouse HRP conjugates were obtained from Bio-Rad (Hercules, CA, USA).

### 4.2. pKAL Compounds

pKAL compounds were extracted from mixed tissues including roots, stems, leaves, and flowers of *Artemisa annua* L. grown at Gaddongsook farm in Jinju, Korea, by Prof. Sung Chul Shin, as previously reported [44]. To isolate pKAL compounds, mixed tissues including roots, stems, leaves, and flowers of *Artemisia annua* L. were lyophilized, ground, and extracted with 70% methanol at 60 °C for 20 h. The extract was filtered through a glass funnel and concentrated at 35 °C using a rotary evaporator. To remove fat components, the concentrated aqueous extract was extracted three times with equal volumes of *n*-hexane and methylene chloride. The filtrate was extracted three times with ethyl acetate to isolate the pKAL compounds and dried over anhydrous magnesium sulfate. pKAL compounds identified via liquid chromatography–tandem mass spectrometry (LC/MS/MS) are as follows: Caffeic acid, Quercetin-3-O-galactoside, Mearnsetin-glucoside, Kaempferol-3-O-glucoside, Ferulic acid, Isorhamnetin-glucoside, Diosmetin-7-O-glucoside, Luteolin-7-O-glucoside, Quercetin, Quercetagetin-3-O-methyl ether, Luteolin, 8-methoxy-kaempferol, Quercetagetin-5,3-di-O-methyl ether, Kaempferol, 3,5-dihydroxy-6,7,4′-trimethoxyflavone, 3,5-dihydroxy-6,7,3′,4′-tetramethoxyflavone, and Isorhamnetin. The HPLC chromatogram of pKAL compounds was presented in a previous report [45]. For experiments, pKAL compounds were dissolved in DMSO solvent at a concentration of 100 mg/mL and stored in a −20 °C freezer until use.

### 4.3. Cell Culture

The HCT116 human colorectal cancer cell line was purchased from Korean Cell Line Bank (KCLB No. 10247). HCT116 cells were cultured in maintenance medium consisting of RPMI medium with L-glutamine (300 mg/L), 25 mM HEPES, 25 mM NaHCO_3_, 1% penicillin/streptomycin, and 10% heat-inactivated FBS (Thermo Fisher Scientific, Grand Island, NY, USA) on a culture dish in a 37 °C incubator supplemented with 5% CO_2_ in a humidified atmosphere. HCT116-OxPt-R cells resistant to 5 μM OxPt were generated in a previous study [31] and maintained with 5 μM OxPt in the same culture condition as HCT116 cells. In this study, HCT116-OxPt-R cells from passage 17 (P17) to P22 were used.

### 4.4. Phase-Contrast Microscopy

The morphology of whole cells (attached and floating cells) was analyzed by phase-contrast microscopy (EVOS XL Core, Thermo Fisher Scientific, Oslo, Norway) in a 10x objective (Inf Plan Achro 10X LWD PH, 0.25 NA/6.9 WD) with 150x amplification.

### 4.5. Cell Viability Analysis

Cells grown on a 24-well dish were incubated with maintenance medium containing 10% CCK-8 reagent for 1.5 h in a 37 °C CO_2_ incubator. The reaction solution (100 μL each) was then transferred to a 96-well dish and was analyzed by measuring the absorbance at OD_450nm_ using a microplate reader (Molecular Devices, SoftMax Pro software version 5).

### 4.6. Western Blot and Densitometry Analysis

Whole cells (attached and floating cells) were extracted with 1x SDS sample buffer and were boiled for 5 min at 95 °C. The resultant proteins were separated using SDS-PAGE and transferred to an NC membrane at 30 mA for 13–15 h. After washing with PBST (0.1% Tween-20, PBS) twice for 1 h, the membrane was blocked for 30 min at RT in blocking buffer (3% skim milk, 0.1% Tween-20, PBS) and then incubated with primary antibody in blocking buffer at 4 °C overnight. The blot was then washed with PBST three times for 10 min and incubated with an HRP-conjugated secondary antibody in blocking buffer for 2 h at RT. After being washed with PBST, immunofluorescent proteins were visualized on X-ray film using the ECL Western blot detection system. For densitometry analysis, Western blot film was scanned at high resolution into TIFF file format. The image was converted into JPEG file format using Photoshop, the image mode was changed to grayscale, and the protein bands were quantified using the ImageJ program (version 1.53k).

### 4.7. Phase-Contrast Microscopy of Trypan Blue-Stained Cells

Trypan blue (negative charged) does not interact with cells without a damaged membrane, and thus trypan blue staining is used to detect dead cells [46,47]. With the medium, the cells were treated with 0.1% trypan blue concentration for 20 min at RT, and were analyzed using phase-contrast light microscopy in a 20x objective (Inf Plan Fluor 20x LWD, 0.45NA/7.1WD) (EVOS XL Core, Thermo Fisher Scientific, Oslo, Norway) with 300x amplification.

### 4.8. Phase-Contrast Microscopy of Hematoxylin-Stained Cells

Hematoxylin (cationic) staining is used to detect the nucleus (DNA, RNA, and acid nucleoprotein) [46]. Cells grown on a 6-well plate were washed with PBS, fixed with 4% formaldehyde solution, washed with PBS, and then stained with hematoxylin solution for 24 h at RT by gently shaking. The cells were washed with PBS and were analyzed in the presence of 90% glycerol/PBS solution via phase-contrast microscopy (EVOS XL Core, Thermo Fisher Scientific) in a 20x objective (Inf Plan Fluor 20x LWD, 0.45 NA/7.1 WD) with 300x amplification.

### 4.9. Bioinformatics Analysis

A gene ontology analysis of proteins regulated by pKAL and β-Lap was performed using the Database for Annotation, Visualization and Integrated Discovery (DAVID) program (v.6.8) via the internet (http://david.ncifcrf.gov/tools.jsp) (accessed on 10 September 2023). String analysis for the protein–protein interaction network was performed using Cytoscape software (v.3.7.1.) via the internet (http://www.cytoscape.org) (accessed on 10 September 2023).

### 4.10. Statistical Analysis

Results for cell viability are presented as mean ± standard deviation of the mean. Statistical significance between the control and sample was determined using Student’s *t*-test. Values of *p* < 0.05 are considered statistically significant.

## Figures and Tables

**Table 1:** Protein information associated with the enhanced anticancer effect of β-Lap by pKAL in HCT116-OxPt-R cells.

Gene Symbol	Protein Name	UniProt ID	Keywords
AKT1	RAC-alpha serine/threonine-protein kinase (AKT1)	P31749	Kinase; Transferase; Apoptosis; Glycogen biosynthesis; Carbohydrate/Glucose/Glycogen metabolism
BECN1	Beclin-1	Q14457	Autophagy; Apoptosis; Cell cycle; Cell division
BIRC5	Baculoviral IAP repeat-containing protein 5 (Survivin)	O15392	Thiol protease inhibitor; Repressor; Apoptosis; Cell cycle; Cell division; Chromosome partition
CASP8	Caspase-8	Q14790	Hydrolase; Thiol protease; Apoptosis
CCNA1	Cyclin-A1 (Cyclin A1)	P78396	Cell cycle; Cell division; Mitosis
CCNB1	G2/mitotic-specific cyclin-B1 (Cyclin B1)	P14635	Cell cycle; Cell division; Mitosis
CCND1	G1/S-specific cyclin-D1 (Cyclin D1)	P24385	Repressor, Cell cycle; Cell division; DNA damage; Transcription
CD44	CD44 antigen (CD44)	P16070	Blood group antigen; Receptor; Cell adhesion
CTNNB1	Catenin beta-1 (β-Catenin)	P35222	Activator; Cell adhesion; Neurogenesis; Transcription; Wnt signaling pathway
EGFR	Epidermal growth factor receptor (EGFR)	P00533	Receptor; Tyrosine-protein kinase; Transferase
H2AX/H2AFX	Histone H2AX (H2AX)	P16104	Cell cycle; DNA damage; DNA recombination; DNA repair; Meiosis
HSP90AA1	Heat shock protein 90-alpha (HSP90α)	P07900	Chaperone; Hydrolase; Stress response
LDHA	L-lactate dehydrogenase A chain (LDHA)	P00338	Oxidoreductase
MAP1LC3B	Microtubule-associated proteins 1A/1B light chain 3B (LC3B)	Q9GZQ8	Autophagy; Ubl conjugation pathway
MAPK3	Mitogen-activated protein kinase 3/p44-ERK1 (ERK1)	P27361	Serine/threonine-protein kinase; Transferase; Apoptosis; Cell cycle
PARP1	Poly [ADP-ribose] polymerase 1 (PARP1)	P09874	Transferase; Apoptosis; DNA damage; DNA repair
PCNA	Proliferating cell nuclear antigen (PCNA)	P12004	DNA damage; DNA repair; DNA replication
PLCG1	Phospholipase C-gamma-1 (PLCγ1)	P19174	Hydrolase; Transducer; Lipid degradation/metabolism
RELA	Nuclear factor NF-kappa-B p65 subunit (NF-κB p65)	Q04206	Activator; Transcription
TERT	Telomerase reverse transcriptase (TERT)	O14746	RNA-directed DNA polymerase; Transferase
TP53	Cellular tumor antigen p53 (p53)	P04637	Activator; Repressor; Apoptosis; Cell cycle; Necrosis

**Table 2:** Gene ontology for proteins associated with the enhanced anticancer effect of β-Lap by pKAL in HCT116-OxPt-R cells. GO_BP, Gene ontology for biological process.

GO_BP_Term	p-Value	Gene symbols
GO:0043066~negative regulation of apoptotic process	5.58 × 10−7	BECN1, CTNNB1, BIRC5, AKT1, TP53, CD44, RELA, EGFR
GO:0001934~positive regulation of protein phosphorylation	2.06 × 10−6	HSP90AA1, CCND1, BIRC5, AKT1, EGFR, MAPK3
GO:0071364~cellular response to epidermal growth factor stimulus	1.21 × 10−5	BECN1, AKT1, PLCG1, EGFR
GO:1900087~positive regulation of G1/S transition of mitotic cell cycle	1.89 × 10−5	CCND1, TERT, AKT1, EGFR
GO:1902895~positive regulation of pri-miRNA transcription from RNA polymerase II promoter	2.12 × 10−5	TERT, TP53, RELA, EGFR
GO:0042981~regulation of apoptotic process	9.91 × 10−5	HSP90AA1, CASP8, BIRC5, AKT1, TP53
GO:0009410~response to xenobiotic stimulus	1.16 × 10−4	BECN1, HSP90AA1, CCND1, CTNNB1, TP53
GO:0006974~cellular response to DNA damage stimulus	2.01 × 10−4	H2AX/H2AFX, CCND1, PARP1, TP53, MAPK3
GO:0000423~macromitophagy	2.09 × 10−4	BECN1, MAP1LC3B, TP53
GO:0044772~mitotic cell cycle phase transition	2.75 × 10−4	CCNA1, CCNB1, CCND1

## Data Availability

Data is contained within the article.

## References

[1] Ding S., Xu S., Fang J., Jiang H. (2020). The Protective Effect of Polyphenols for Colorectal Cancer. Front. Immunol..

[2] Bracci L., Fabbri A., Del Corno M., Conti L. (2021). Dietary Polyphenols: Promising Adjuvants for Colorectal Cancer Therapies. Cancers.

[3] Dana P.M., Sadoughi F., Asemi Z., Yousefi B. (2022). The role of polyphenols in overcoming cancer drug resistance: A comprehensive review. Cell. Mol. Biol. Lett..

[4] Di Lorenzo C., Colombo F., Biella S., Stockley C., Restani P. (2021). Polyphenols and Human Health: The Role of Bioavailability. Nutrients.

[5] Li L., Jin P., Guan Y., Luo M., Wang Y., He B., Li B., He K., Cao J., Huang C. (2022). Exploiting Polyphenol-Mediated Redox Reorientation in Cancer Therapy. Pharmaceuticals.

[6] Khan H., Reale M., Ullah H., Sureda A., Tejada S., Wang Y., Zhang Z.J., Xiao J. (2020). Anti-cancer effects of polyphenols via targeting p53 signaling pathway: Updates and future directions. Biotechnol. Adv..

[7] Jung E.J., Lee W.S., Paramanantham A., Kim H.J., Shin S.C., Kim G.S., Jung J.M., Ryu C.H., Hong S.C., Chung K.H. (2020). p53 Enhances *Artemisia annua* L. Polyphenols-Induced Cell Death through Upregulation of p53-Dependent Targets and Cleavage of PARP1 and Lamin A/C in HCT116 Colorectal Cancer Cells. Int. J. Mol. Sci..

[8] Jung E.J., Paramanantham A., Kim H.J., Shin S.C., Kim G.S., Jung J.M., Ryu C.H., Hong S.C., Chung K.H., Kim C.W. (2021). *Artemisia annua* L. Polyphenol-Induced Cell Death Is ROS-Independently Enhanced by Inhibition of JNK in HCT116 Colorectal Cancer Cells. Int. J. Mol. Sci..

[9] Dzobo K., Sinkala M. (2021). Cancer Stem Cell Marker CD44 Plays Multiple Key Roles in Human Cancers: Immune Suppression/Evasion, Drug Resistance, Epithelial-Mesenchymal Transition, and Metastasis. OMICS.

[10] Sethi K., Sarkar S., Das S., Rajput S., Mazumder A., Roy B., Patra S., Mohanty B., El-Naggar A.K., Mandal M. (2011). Expressions of CK-19, NF-kappaB, E-cadherin, beta-catenin and EGFR as diagnostic and prognostic markers by immunohistochemical analysis in thyroid carcinoma. J. Exp. Ther. Oncol..

[11] Gomes C.L., de Albuquerque Wanderley Sales V., Gomes de Melo C., Ferreira da Silva R.M., Vicente Nishimura R.H., Rolim L.A., Rolim Neto P.J. (2021). Beta-lapachone: Natural occurrence, physicochemical properties, biological activities, toxicity and synthesis. Phytochemistry.

[12] Gong Q., Hu J., Wang P., Li X., Zhang X. (2021). A comprehensive review on beta-lapachone: Mechanisms, structural modifications, and therapeutic potentials. Eur. J. Med. Chem..

[13] Sunassee S.N., Veale C.G., Shunmoogam-Gounden N., Osoniyi O., Hendricks D.T., Caira M.R., de la Mare J.A., Edkins A.L., Pinto A.V., da Silva Junior E.N. (2013). Cytotoxicity of lapachol, beta-lapachone and related synthetic 1,4-naphthoquinones against oesophageal cancer cells. Eur. J. Med. Chem..

[14] Kim I., Kim H., Ro J., Jo K., Karki S., Khadka P., Yun G., Lee J. (2015). Preclinical Pharmacokinetic Evaluation of beta-Lapachone: Characteristics of Oral Bioavailability and First-Pass Metabolism in Rats. Biomol. Ther..

[15] Pink J.J., Planchon S.M., Tagliarino C., Varnes M.E., Siegel D., Boothman D.A. (2000). NAD(P)H:Quinone oxidoreductase activity is the principal determinant of beta-lapachone cytotoxicity. J. Biol. Chem..

[16] Kim D.W., Cho J.Y. (2018). NQO1 is Required for beta-Lapachone-Mediated Downregulation of Breast-Cancer Stem-Cell Activity. Int. J. Mol. Sci..

[17] Zhao W., Jiang L., Fang T., Fang F., Liu Y., Zhao Y., You Y., Zhou H., Su X., Wang J. (2021). Beta-Lapachone Selectively Kills Hepatocellular Carcinoma Cells by Targeting NQO1 to Induce Extensive DNA Damage and PARP1 Hyperactivation. Front. Oncol..

[18] Yu H.Y., Kim S.O., Jin C.Y., Kim G.Y., Kim W.J., Yoo Y.H., Choi Y.H. (2014). Beta-lapachone-Induced Apoptosis of Human Gastric Carcinoma AGS Cells Is Caspase-Dependent and Regulated by the PI3K/Akt Pathway. Biomol. Ther..

[19] Choi B.T., Cheong J., Choi Y.H. (2003). Beta-Lapachone-induced apoptosis is associated with activation of caspase-3 and inactivation of NF-kappaB in human colon cancer HCT-116 cells. Anticancer Drugs.

[20] Park E.J., Min K.J., Lee T.J., Yoo Y.H., Kim Y.S., Kwon T.K. (2014). Beta-Lapachone induces programmed necrosis through the RIP1-PARP-AIF-dependent pathway in human hepatocellular carcinoma SK-Hep1 cells. Cell Death Dis..

[21] Moon D.O., Kang C.H., Kim M.O., Jeon Y.J., Lee J.D., Choi Y.H., Kim G.Y. (2010). Beta-lapachone (LAPA) decreases cell viability and telomerase activity in leukemia cells: Suppression of telomerase activity by LAPA. J. Med. Food.

[22] Li C.J., Averboukh L., Pardee A.B. (1993). Beta-Lapachone, a novel DNA topoisomerase I inhibitor with a mode of action different from camptothecin. J. Biol. Chem..

[23] Krishnan P., Bastow K.F. (2001). Novel mechanism of cellular DNA topoisomerase II inhibition by the pyranonaphthoquinone derivatives alpha-lapachone and beta-lapachone. Cancer Chemother. Pharmacol..

[24] Park E.J., Choi K.S., Kwon T.K. (2011). Beta-Lapachone-induced reactive oxygen species (ROS) generation mediates autophagic cell death in glioma U87 MG cells. Chem. Biol. Interact..

[25] Planchon S.M., Wuerzberger S., Frydman B., Witiak D.T., Hutson P., Church D.R., Wilding G., Boothman D.A. (1995). Beta-lapachone-mediated apoptosis in human promyelocytic leukemia (HL-60) and human prostate cancer cells: A p53-independent response. Cancer Res..

[26] Huang L., Pardee A.B. (1999). Beta-lapachone induces cell cycle arrest and apoptosis in human colon cancer cells. Mol. Med..

[27] D’Anneo A., Augello G., Santulli A., Giuliano M., di Fiore R., Messina C., Tesoriere G., Vento R. (2010). Paclitaxel and beta-lapachone synergistically induce apoptosis in human retinoblastoma Y79 cells by downregulating the levels of phospho-Akt. J. Cell Physiol..

[28] Kim E.J., Ji I.M., Ahn K.J., Choi E.K., Park H.J., Lim B.U., Song C.W., Park H.J. (2005). Synergistic effect of ionizing radiation and beta-Lapachone against RKO human colon adenocarcinoma cells. Cancer Res. Treat..

[29] Falcone A., Ricci S., Brunetti I., Pfanner E., Allegrini G., Barbara C., Crinò L., Benedetti G., Evangelista W., Fanchini L. (2007). Phase III trial of infusional fluorouracil, leucovorin, oxaliplatin, and irinotecan (FOLFOXIRI) compared with infusional fluorouracil, leucovorin, and irinotecan (FOLFIRI) as first-line treatment for metastatic colorectal cancer: The Gruppo Oncologico Nord Ovest. J. Clin. Oncol..

[30] Cassidy J., Clarke S., Díaz-Rubio E., Scheithauer W., Figer A., Wong R., Koski S., Lichinitser M., Yang T.S., Rivera F. (2008). Randomized phase III study of capecitabine plus oxaliplatin compared with fluorouracil/folinic acid plus oxaliplatin as first-line therapy for metastatic colorectal cancer. J. Clin. Oncol..

[31] Jung E.J., Kim H.J., Shin S.C., Kim G.S., Jung J.M., Hong S.C., Kim C.W., Lee W.S. (2023). Beta-Lapachone Exerts Anticancer Effects by Downregulating p53, Lys-Acetylated Proteins, TrkA, p38 MAPK, SOD1, Caspase-2, CD44 and NPM in Oxaliplatin-Resistant HCT116 Colorectal Cancer Cells. Int. J. Mol. Sci..

[32] Feng X., Cao S., Qiu F., Zhang B. (2020). Traditional application and modern pharmacological research of *Artemisia annua* L.. Pharmacol. Ther..

[33] Efferth T. (2017). Cancer combination therapies with artemisinin-type drugs. Biochem. Pharmacol..

[34] Kadioglu O., Chan A., Cong Ling Qiu A., Wong V.K.W., Colligs V., Wecklein S., Freund-Henni Rached H., Efferth T., Hsiao W.W. (2017). Artemisinin Derivatives Target Topoisomerase 1 and Cause DNA Damage in Silico and in Vitro. Front. Pharmacol..

[35] Li P.C., Lam E., Roos W.P., Zdzienicka M.Z., Kaina B., Efferth T. (2008). Artesunate derived from traditional Chinese medicine induces DNA damage and repair. Cancer Res..

[36] Berdelle N., Nikolova T., Quiros S., Efferth T., Kaina B. (2011). Artesunate induces oxidative DNA damage, sustained DNA double-strand breaks, and the ATM/ATR damage response in cancer cells. Mol. Cancer Ther..

[37] Michaelsen F.W., Saeed M.E., Schwarzkopf J., Efferth T. (2015). Activity of Artemisia annua and artemisinin derivatives, in prostate carcinoma. Phytomedicine.

[38] Lang S.J., Schmiech M., Hafner S., Paetz C., Steinborn C., Huber R., Gaafary M.E., Werner K., Schmidt C.Q., Syrovets T. (2019). Antitumor activity of an Artemisia annua herbal preparation and identification of active ingredients. Phytomedicine.

[39] Zhang Y., Liu K., Yan C., Yin Y., He S., Qiu L., Li G. (2022). Natural Polyphenols for Treatment of Colorectal Cancer. Molecules.

[40] Vuoso D.C., D’Angelo S., Ferraro R., Caserta S., Guido S., Cammarota M., Porcelli M., Cacciapuoti G. (2020). Annurca apple polyphenol extract promotes mesenchymal-to-epithelial transition and inhibits migration in triple-negative breast cancer cells through ROS/JNK signaling. Sci. Rep..

[41] Ko Y.S., Lee W.S., Panchanathan R., Joo Y.N., Choi Y.H., Kim G.S., Jung J.M., Ryu C.H., Shin S.C., Kim H.J. (2016). Polyphenols from *Artemisia annua* L. Inhibit Adhesion and EMT of Highly Metastatic Breast Cancer Cells MDA-MB-231. Phytother. Res..

[42] Vladu A.F., Ficai D., Ene A.G., Ficai A. (2022). Combination Therapy Using Polyphenols: An Efficient Way to Improve Antitumoral Activity and Reduce Resistance. Int. J. Mol. Sci..

[43] Ko Y.S., Jung E.J., Go S.I., Jeong B.K., Kim G.S., Jung J.M., Hong S.C., Kim C.W., Kim H.J., Lee W.S. (2020). Polyphenols Extracted from *Artemisia annua* L. Exhibit Anti-Cancer Effects on Radio-Resistant MDA-MB-231 Human Breast Cancer Cells by Suppressing Stem Cell Phenotype, beta-Catenin, and MMP-9. Molecules.

[44] Song Y., Desta K.T., Kim G.S., Lee S.J., Lee W.S., Kim Y.H., Jin J.S., Abd El-Aty A.M., Shin H.C., Shim J.H. (2016). Polyphenolic profile and antioxidant effects of various parts of *Artemisia annua* L.. Biomed. Chromatogr..

[45] Jung E.J., Paramanantham A., Kim H.J., Shin S.C., Kim G.S., Jung J.M., Hong S.C., Chung K.H., Kim C.W., Lee W.S. (2022). Identification of Growth Factors, Cytokines and Mediators Regulated by *Artemisia annua* L. Polyphenols (pKAL) in HCT116 Colorectal Cancer Cells: TGF-beta1 and NGF-beta Attenuate pKAL-Induced Anticancer Effects via NF-kappaB p65 Upregulation. Int. J. Mol. Sci..

[46] Atale N., Gupta S., Yadav U.C., Rani V. (2014). Cell-death assessment by fluorescent and nonfluorescent cytosolic and nuclear staining techniques. J. Microsc..

[47] Crowley L.C., Marfell B.J., Christensen M.E., Waterhouse N.J. (2016). Measuring Cell Death by Trypan Blue Uptake and Light Microscopy. Cold Spring Harb. Protoc..

